# Association of Internal Mammary Artery Flow with Different Comorbidities and Post-Coronary Artery Bypass Graft Complications

**DOI:** 10.7759/cureus.1584

**Published:** 2017-08-20

**Authors:** Mudassir Iqbal Dar, Asim Hassan Dar, Muhammad Bilal, Mansoor Ahmad, Abdul Haseeb

**Affiliations:** 1 Dow University of Health Sciences, Karachi, Pakistan; 2 Institute for Biology, Otto Von Guericke University; 3 Department of Medicine, Dow University of Health Sciences, Karachi, Pakistan; 4 Department of Pharmacy, Karachi University, Karachi University

**Keywords:** cabg, internal mammary artery, ima flow, postoperative complications

## Abstract

Objective

The internal mammary artery (IMA) is commonly used arterial graft for coronary artery bypass surgery (CABG). The IMA has a better patency and survival. This study considers different comorbidities and conditions where IMA flow may be low. Therefore, the main objective is to determine the flow of IMA in different diseases, its relation to gender, age, and in different blood groups in order to prevent possible complications.

Methods

A prospective study was conducted at the Cardiac Surgery Unit, Civil Hospital Karachi from January 2013 to December 2015. The data of 158 patients who underwent primary, isolated, and CABG surgery was collected. Free flow of IMA was measured immediately after harvesting for 30 seconds within a syringe, and its relationship with different diseases and conditions was examined. Data was collected using a structured questionnaire from the patients' medical record files and it was later analyzed and entered into the Statistical Package for Social Sciences (SPSS) software, V17 (IBM SPSS Statistics, Armonk, NY).

Results

The mean flow was 11.6 ± 9.6 ml. There was no difference in flow related to gender, diabetes, smoking, renal disease, and chronic obstructive pulmonary disease (COPD). There was significantly higher flow in the age group of 50 to 60 years (p = 0.002), hypertensive patients (p = 0.016), patients with liver disease (p = 0.001), BMI > 30 (p = 0.041), and the blood group AB+ (p = 0.02). The atrial fibrillation and readmissions were higher in patients who had low flow. Low flow IMA, which was used on left anterior descending (LAD) artery stenosis patients, showed a significantly higher need of pharmacological and mechanical support.

Conclusion

IMA should be used carefully in patients where its flow is low; conditions must be analyzed where flow may be low to avoid complications. Further studies are warranted.

## Introduction

The left internal mammary artery (IMA) is the most frequently used arterial graft for myocardial revascularization. Its superiority over saphenous vein graft leaves little doubt because of the tight endothelial junction and production of biochemical substances like nitric oxide and antithrombotic factors, and resistance to the generation of selectins and other molecules, which may be the reason for IMA being resistant to developing atherosclerosis [1]. In coronary artery bypass surgery (CABG) where IMA is used for grafting, this is particularly important for patients with diabetes mellitus because survival is significantly greater in patients with diabetes after surgery as compared with percutaneous transluminal angioplasty [2]. Operative mortality is also reduced with the use of IMA in CABG surgery [3]. The use of bilateral IMAs is also associated with a further increase in long-term survival and freedom from myocardial infarction, recurrence of angina, percutaneous re-intervention, and repeat operation [4]. IMA has other advantages, such as its ability to dilate in response to an increased myocardial blood flow demand [5]. 

IMA is an ideal conduit for coronary artery bypass surgery; however, there are a few disadvantages associated with its use. IMA flow through graft is often less than vein graft in the early postoperative period, owing to resistance by the IMA wall [6]. Low IMA graft flow may produce early postoperative hemodynamic collapse, which results in high mortality and morbidity [7]. Some patients developed refractory low cardiac output syndrome and ventricular dysrhythmia after CABG surgery, which is generally considered to be related to the insufficient flow in the IMA, and these patients needed urgent reoperation [8]. About 2% of the patients developed malperfusion, which is defined as an imbalance between demand and nutritional support through the IMA. The mortality in malperfusion syndrome is up to 66% [9]. Failure of IMA is common and also associated with higher rates of repeat revascularization. Moreover, moderate left anterior descending (LAD) artery stenosis patients and subjects with an additional bypass graft to the diagonal branch are susceptible to increased risk of IMA failure [10]. Blood transfusion and increased postoperative bleeding are also associated with the use of IMA [10]. The complications altogether are much less than its benefits, but they cannot be ignored as they are associated with higher mortality and morbidity. Therefore, on the basis of these observations, a study was designed to review the flow of IMA in different disease states as well as its relation to gender, age, and in different blood groups in order to provide useful information in determining a preoperative plan and to prevent possible complications in these groups of patients. Consequently, complications may be predicted and mortality/morbidity can be reduced and prevented.  

## Materials and methods

This study was conducted in a leading public institute of cardiac surgery, the Civil Hospital Karachi, over a period of two years from January 2013 to December 2015. Data were collected prospectively by convenience sampling for 158 consecutive patients who underwent primary, isolated, and elective coronary artery bypass surgery (CABG). Emergency surgery and urgent referral for surgery were excluded; patients who underwent redo-coronary artery bypass surgery, off-pump surgery, and CABG with valve surgery were also excluded.

The study was approved by the Institutional Review Board of Dow University of Health Sciences (# IRB/DUHS/2013/216/081). Before obtaining data, all patients were briefly explained about the purpose and outcomes of the study, and an informed consent was obtained from each participant.

The data collection tool consisted of a questionnaire which comprised of demographic details of subjects, associated comorbidities, IMA flow, and postoperative (CABG) complications in study subjects. The obtained data was entered on the Statistical Package for the Social Sciences (SPSS) (V17) software (IBM SPSS Statistics, Armonk, NY) and was further analyzed by using the same tool. We computed frequencies and percentages for all quantitative variables. 

## Results

A total of 158 patients were prospectively evaluated; 129 (81.6%) were male and 29 (18.4%) were female. The mean age was 52 with standard deviation of ± 8 years. Age groups were divided in following groups; eight patients (5%) were 30 - 40 years old, 47 patients (29.7%) were 40 - 50 years old, 74 patients (46.8%) were 50 - 60 years old, 21 patients (13.3%) were 60 - 70 years old, and eight patients (5%) were 70 or more years old.  Diabetes mellitus was present in 66 subjects (41.8%) and hypertension was present in 116 (73.4%) patients. Obesity (body mass index exceeding 30 kg/m2) was present in 47 (29.7%) patients, the mean weight was 72.5 ± 12.4 kg, mean height was 164 ± 9 cm, and mean BMI was 26.9 ± 4.6 kg/m2. BMI less than 20 was present in four (2.5%), from 21 to 24 was present in 53 (33.5%), and from 25 to 29 present in 54 subjects (54.2%). Smoking was present in 77 (48.7%) patients. The Canadian Cardiovascular Society classification of angina (CCSA) Class II was present in 35 patients (22.2%), Class III in 102 patients (64.6%), and Class IV in 21 (13.3%) patients. Furthermore, New York Heart Association (NYHA) Class I was present in six patients (3.8%), Class II in 117 patients (74.1%), Class III in 29 patients (18.4%), and Class IV in six participants (3.8%). Renal impairment (creatinine more than 2 dl/ml) was present in nine patients (5.7%), a carotid bruit was present in two (1.3%), COPD in five (3.2%), stroke was present in five (3.2%), liver disease was present in eight (5.1%), and a history of tuberculosis was present in three (1.9%) patients. Preoperative myocardial infarction (MI) was present in 114 (72%) patients. Left ventricular function was normal (more than 60%) in 30 (19%) patients, moderate (30 to 60%) in 107 (67.7%) patients, and poor (less than 30%) in 21 (13.3%) patients. A left main coronary artery lesion was present in nine (5.6%) patients. The blood groups among the study patients were A+ (n = 51) (32.3%), AB+ (n = 17) (10.8%), B+ (n = 41) (25/9%), O+ (n = 39) (24.7%), O- (n = 4) (2.5%), B- (n = 3) (1.9%), AB- (n = 2) (1.3%), and A- (n = 1) (.6%).

Before observing the flow of IMA in 158 patients, the mean heart rate with standard deviation was 83 ± 17 per minutes, the systolic blood pressure was 104 ± 14 mm of Hg, the diastolic blood pressure was 64 ± 10 mm of Hg, the mean arterial pressure was 76 ± 12 mm of Hg, and the central venous pressure was 7 ± 3 mm of Hg. The room temperature was also observed and found to be 31 ± 5º C at the time of the first study. The mean volume of blood expelled by the 158 patients in 30 seconds was 11.6 ± 9.7 mls. The minimum was 0 ml per 30 seconds, and the maximum was 63 ml in 30 seconds. Forty (25%) patients had less than 5 mls of blood expelled in 30 seconds, 5 to 10 ml per 30 seconds was present in 58 (37%) patients, 10 to 20 ml was present in 35 (22%) patients, 20 to 30 mls in another 17 (11%) patients, and more than 30 ml in only eight (5%) patients. Therefore, the maximum number of patients had their IMA flow from 5 to 10 ml per 30 seconds

Table 1 shows the different age groups in relation to IMA flow.

**Table 1:** Internal Mammary Artery Flow in Different Age Groups

Age of patients (in years)	Mean flow with standard deviation	*p* = value
30 - 40 (n = 8)	12.5 ± 7.5	*p *= .985
40 - 50 (n = 47)	9.5 ± 6.7 low as compared to group 3	*p *= .002
50 - 60 (n = 74)	13.7 ± 12 high as compared to group 2&4	*p *= .002
60 - 70 (n = 21)	9.2 ± 5.8 low as compared to group 3	*p *= .002
> 70 (n = 8)	11.5 ± 5	*p *= .923

Table 2 compares the IMA flow in different genders and people with other preoperative comorbidities. Although the flow in male patients was higher at 11.9 ± 9.6 ml per 30 seconds as compared to the females where the flow was 9.2 ± 7 ml per 30 seconds, there was no significant difference. The diabetic patients also had a higher IMA flow (12.2 ± 8 ml per 30 seconds) as compared to non-diabetics (10.8 ± 9 ml per 30 seconds), but there was no significant difference. Patients with renal impairment had an IMA flow of 13.6 ± 7.5 ml per 30 seconds, and the patients with normal kidney function had a flow of 11.3 ± 9 ml per 30 seconds with no significant difference. Patients with COPD showed an IMA flow of 15 ± 8 ml per 30 seconds, and without COPD, the flow was 11 ± 9 ml per 30 seconds with no significant difference. Patients with a carotid bruit had an IMA flow of 14 ± 6 ml per 30 seconds, and with those with no bruit had a flow of 11 ± 9 ml per 30 seconds. Patients with a history of tuberculosis had a lower IMA flow of 7 ± 4 ml per 30 seconds as compared to non-tuberculosis patients at 11.5 ± 9 ml per 30 seconds.

**Table 2:** Internal Mammary Artery Flow in Different Preoperative Conditions BMI: body mass index; COPD: chronic obstructive pulmonary disease; IMA: internal mammary artery; LV: left ventricular

Variables	Mean IMA flow in ml with standard deviation	*p* value
Male/ Female	11.9 ± 9.6 / 9.2 ± 7	*p = .*097
Diabetic/ Non-diabetic	12.2 ± 8 / 10.8 ± 9	*p = .*388
Hypertensive/ Non-hypertensive	12.3 ± 8.4 / 9 ± 6	*p *= 0.016
Renal impairment/ Normal kidney function	13.6 ± 7.5 / 11.3 ± 9.3	*p *= .411
BMI > 30 / BMI <30	13.6 ± 9.6 / 10.3 ± 8.9	*p *= 0.041
Liver disease/ No liver disease	20.3 ± 21 / 11 ± 8	*p *= .005
COPD/ No COPD	15 ± 8 / 11 ± 9	*p *= .340
Smoking/ Non smoking	10.8 ± 8 / 12.1 ± 11	*p *= .404
Normal LV function / Moderate LV function / Poor LV function	13.4 ± 8.8 / 11.6 ± 9.8 / 10.3 ± 5.9	*p *= .398 / *p *= .245 / *p *= .211

Table 3 compares the IMA flow in different blood groups.The highest flow was 17 ± 9.6 ml per 30 seconds with p value of 0.02 associated with the AB+ blood group. The lowest flow was 8 ± 5 ml per 30 seconds associated with the B- blood group but the result was not significant.

**Table 3:** Internal Mammary Artery (IMA) Flow in Different Blood Groups

Blood groups	Mean IMA flow with standard deviation.	*p *= value
A+	11.5 ± 10	*p *= 0.990
B+	11.1 ± 7.8	*p *= 0.990
AB+	17 ± 9.6	*p* = 0.02
O+	10 ± 11	*p *= 0.998
A-	24	- (one patient only)
AB-	15. ± 13	*p *= 0.997
O-	11.5 ± 3	*p *= 1
B-	8 ± 5	*p *= .995

Relationship with smoking showed that the flow of non-smokers was 12 ± 11 ml per 30 seconds, higher than smokers (10.8 ± 8 ml per 30 seconds), but it did not reach significant levels. As far as obesity and high blood pressure was concerned, the patients with high blood pressure had a higher flow of 12.3 ± 8.4 ml per 30 seconds than non-hypertensive 9 ± 6 ml per 30 seconds, showing a significantly higher flow in hypertensives with a p value = 0.016. There was also a higher IMA flow in patients with a higher BMI. Patients with a BMI less than 20 had an IMA flow of 4.7 ± 2 ml per 30 seconds; from 21 to 24, the flow was 9.29 ± 7.6 ml per 30 seconds, BMIs of 25 to 29 had an IMA flow of 12.4 ± 10.5 ml per 30 seconds, and patients with a BMI more than 30 showed a flow of 13.4 ± 10.6 ml per 30 seconds. Therefore, patients with a BMI more the 30 kg/m2 had significantly higher flow than patients with a BMI less than 20 and those ranging from 21 to 24 (p = 0.001 and 0.017, respectively). Patients with established chronic liver disease with a positive hepatitis B or C marker had a significantly higher flow of 20.3 ± 21 ml per 30 seconds as compared to non-reactive liver disease patients where the flow was 11 ± 8 ml per 30 seconds with a p value = 0.005. Patients with different left ventricular functions did not show any significant differences in the IMA flow.

IMA was employed as a graft for LAD artery stenosis. Patients with low IMA flow, despite the application of a topical vasodilator, developed a significantly greater number of low cardiac output syndromes and required more inotropic support and intra-aortic balloon pump placement. The incidence of atrial fibrillation was also high. One patient expired, and there were more readmissions, although not reached to significant levels. The need of an intra-aortic balloon pump (IABP) and readmission were directly related to low flow IMA, although the incidence of atrial fibrillation was high in low flow IMA but did not reach significant levels.

The overall mortality was 3.8% (six out of 158). Low cardiac output syndrome developed in 10% (n = 16) of the patients, five (3%) patients needed an intra-aortic balloon pump, and 11 (7%) developed atrial fibrillation. The mean ICU stay was 2.3 ± 0.8 days, the mean ward stay was 4.0 ± 2.2 days, and the mean intubation time was 6.0 ± 3.6 hours. Five (3%) patients were readmitted for different reasons, six (3.8%) needed reopening for bleeding, four (2.5%) developed chest wound infection, three (1.9%) patients developed stroke after surgery, and two (1.2%) developed postoperative acute kidney injury, which settled with conservative treatment. Endarterectomy was needed in six (3.8%) patients; one patient needed all vessels to undergo endarterectomy and died on the first postoperative day, and four patients were needed to be put urgently on bypass after harvesting the IMA because of the persistently low blood pressure. Two patients needed chest tube insertion postoperatively and needed to stay for a longer duration in the hospital. Three patients developed malperfusion syndrome immediately after surgery and needed one extra vein graft to LAD, of which one of them expired. Moreover, one patient developed subclavian artery damage during harvesting the IMA, one patient needed rewiring of the sternal wound, three patients showed deep intramyocardial LAD artery stenosis, and two patients developed ST elevation before undergoing bypass.

## Discussion

The left internal mammary artery is routinely used for the left anterior descending coronary artery as a part of an elective coronary artery bypass grafting procedure due to its excellent long-term patency and long-term survival in CABG patients [1]. Several previous studies have shown that a careful harvesting technique for the IMA is associated with the good flow and only occasionally topical vasodilators are used to overcome the spasm, while none of them emphasize on its relationship with different diseases and conditions [11-12].

This study has highlighted the effect of different diseases and conditions on IMA flow, and therefore, predictions can be made regarding which patients will get more benefit from harvesting the IMA. The interesting finding in this study is that there is a difference in IMA flow in patients with different blood groups, showing a significantly higher flow in the AB+ blood group patients. The patients with AB- and A- also had high IMA blood flow; however, as the number of patients was less, it did not reach significant levels. The lowest flow was observed in the B- group patients.

In our study, the IMA flow was higher in the hypertensive patients than in the non-hypertensive patients, with a significant difference in flow. This high flow pattern might be related to some antihypertensive medicines that the patient was taking before surgery, as shown in other studies, as these drugs have vasodilatory effects [12].

We also found that patients suffering from hepatic disease with positive hepatitis B or C virus also had an increased flow in the internal mammary artery. This may be related to patients with liver disease who have a higher synthesis of nitric oxide (NO), which is a potent vasodilator. Nitric oxide, a potent vasodilator, is synthesized and released from peripheral blood vessels in the body [13].

We also found an age-wise difference in IMA flow; the highest IMA flow was in patients who were in the 50 to 60 years of age group and the lowest in the age group of 60 to 70 years old. No study has so far shown the flow of IMA in different age groups. Only some studies showed less use of the IMA in the patient age group more than 70 years old [14].

Our study also showed that there was a significantly higher IMA flow in obese patients with a BMI greater than 30 kg/m2. Studies which have recommended against usage of IMA in obese patients do mention about the higher flow but observed a higher incidence of mediastinitis, postoperative blood loss, and pneumothorax [15]. These factors are the primary reasons for not preferring bilateral IMA harvesting in obese patients.

The IMA flow in males and females are statistically the same in our study. In the 129 males, the flow was 11.9 ± 9.6 ml per 30 seconds. Of the 29 females, it was 9.2 ± 7 ml in 30 seconds, showing a lower flow in females. However, the difference was non-significant. One study showed that the flow in males was 33.7 ± 2 and in females was 29.4 ± 3, which also indicated a lower flow in females [16].

The IMA flow in smokers was also the same and without any significant difference in our study. Although the flow was higher in non-smokers, it did not reach a significant level. There are studies which show a poor response to relaxation of the IMA segment harvested in smokers [17]. There are also studies showing reduced peripheral microvascular responses to endothelial and smooth muscle cell stimulation and showed a generalized microvascular vasomotor dysfunction in smokers [18].

IMA flow was higher in patients with good left ventricular function, and as the ventricular function reduces, IMA flow also reduces in proportion. However, they did not reach a significant level. The reason might be the gradual rather than sudden change in left ventricular function. This point can be further evaluated in future studies. 

IMA flow was not different in the patients who developed complications, such as low cardiac output syndrome, atrial fibrillation, or the patients who died. There was a significant association of use of low flow IMA and higher need of IABP postoperatively. The readmission rate was also high in patients with low flow IMA. 

Low flow in IMA less than 5 ml in 30 seconds was present in 40 patients. In this low flow group, we used IMA in 27 patients; in the rest of the 13 patients, only a vein graft was used on the LAD artery stenosis. There were very interesting findings when we used the IMA in the low flow group; the response of the operation was not as good as where it was not employed. In patients where the IMA was used with low flow, there was a significantly higher rate of IABP used (p = 0.028), higher doses of inotropic drugs used (p = 0.004), and a higher number of low cardiac output syndromes (p = 0.022). There were also more atrial fibrillations, more deaths, and more readmissions after discharge where low flow IMA was used, but it did not reach significant levels. Therefore, use of an IMA should be necessary for all patients, but different comorbidities should be taken into consideration before its employment.

## Conclusions

There is a direct relationship of high IMA flow with BMI > 30, hypertension, liver disease, and the age group from 50 to 60 years old where the flow is high and its use should be encouraged as it is associated with fewer postoperative complications. Other conditions in which IMA flow may be low must be considered cautionary, such as different age groups, different blood groups, and in normotensive patients, due to the incidence of severe postoperative complications. Further studies are warranted to verify the conditions where due precautions should be taken when using the IMA.

## References

[1] Otsuka F, Yahagi K, Sakakura K, Virmani R (2013). Why is the mammary artery so special and what protects it from atherosclerosis?. Ann Cardiothorac Surg.

[2] Farkouh ME, Domanski M, Fuster V (2014). Comparison of CABG versus PCI outcomes in diabetic, multivessel coronary artery disease patients with and without acute coronary syndromes. Circulation.

[3] Kaufer E, Factor SM, Frame R, Brodman RF (1997). Pathology of the radial and internal thoracic arteries used as coronary artery bypass grafts. Ann Thorac Surg.

[4] Weiss AJ, Zhao S, Tian DH (2013). A meta-analysis comparing bilateral internal mammary artery with left internal mammary artery for coronary artery bypass grafting. Ann Cardiothorac Surg.

[5] Takayama T, Suma H, Wanibuchi Y (1992). Physiological and pharmacological responses of arterial graft flow after coronary artery bypass grafting measured with an implantable Doppler miniprobe. Circulation.

[6] Dobrin P, Canfield T, Moran J (1977). Coronary artery bypass. The physiological basis for differences in flow with internal mammary artery and saphenous vein grafts. J Thorac Cardiovasc Surg.

[7] Vajtai P, Ravischaundran PS, Fessler CL (1992). Inadequate internal mammary artery graft as a cause of postoperative ischemia: incidence, diagnosis and management. Eur J Cardiothorac Surg.

[8] Sarabu MR, Mc Clung JA, Fass A, Reed GE (1987). Early postoperative spasm in left internal mammary artery bypass grafts. Ann Thorac Surg.

[9] Massoudy P, Kim YY, Cetin M (2004). Internal thoracic artery malperfusion: fast decision for a additional vein graft has impact on patient outcome. Ann Thorac Surg.

[10] Harskamp RE, Alexander JH, Ferguson TB Jr (2016). Frequency and predictors of internal mammary artery graft failure and subsequent clinical outcomes: Insights From the Project of Ex-vivo Vein Graft Engineering via Transfection (PREVENT) IV Trial. Circulation.

[11] Mand'ák J, Lonský V, Dominik J (1999). Topical use of aprotinin in coronary artery bypass surgery. Acta Medica (Hradec Kralove).

[12] Sarikaya S, Onk A, Boztosun B (2014). The effect of nebivolol on internal mammary artery blood flow during coronary artery bypass graft surgery. Perfusion.

[13] Deshmukh A, More UK, Tilak MA (2013). Role of nitric oxide in liver cirrhosis. Indian J Basic Appl Med Res.

[14] Karthik S, Srinivasan AK, Grayson AD (2004). Use of the left internal mammary artery to the left anterior descending artery: a study of patient characteristics, effect on morbidity and mortality and reasons for non-usage. Ann Thorac Surg.

[15] Hegazy YY, Hassanein W, Ennker J (2017). Use of bilateral internal mammary artery grafting in different degrees of obesity. Thorac Cardiovasc Surg.

[16] Kjaergard HK, Irmukhamedov A, Christensen JB, Schmidt TA (2004). Flow in coronary bypass conduits on-pump and off-pump. Ann Thorac Surg.

[17] Muir AD, McKeown PP, Bayraktutan U (2010). Role of gender, smoking profile, hypertension, and diabetes on saphenous vein and internal mammary artery endothelial relaxation in patients with coronary artery bypass grafting. Oxid Med Cell Longev.

[18] Edvinsson ML, Andersson SE, Xu CB, Edvinsson L (2008). Cigarette smoking leads to reduced relaxant responses of the cutaneous microcirculation. Vasc Health Risk Manag.

